# Applications of social theories of learning in health professions education programs: A scoping review

**DOI:** 10.3389/fmed.2022.912751

**Published:** 2022-07-28

**Authors:** Banan Mukhalalati, Sara Elshami, Myriam Eljaam, Farhat Naz Hussain, Abdel Hakim Bishawi

**Affiliations:** ^1^Clinical Pharmacy and Practice Department, College of Pharmacy, QU Health, Qatar University, Doha, Qatar; ^2^Pharmaceutical Sciences Department, College of Pharmacy, QU Health, Qatar University, Doha, Qatar; ^3^Research and Instruction Section, Library Department, Qatar University, Doha, Qatar

**Keywords:** social learning theory, social cognitive theory, communities of practice, health professions education, teaching, assessment, curriculum

## Abstract

**Introduction:**

In health professions education (HPE), acknowledging and understanding the theories behind the learning process is important in optimizing learning environments, enhancing efficiency, and harmonizing the education system. Hence, it is argued that learning theories should influence educational curricula, interventions planning, implementation, and evaluation in health professions education programs (HPEPs). However, learning theories are not regularly and consistently implemented in educational practices, partly due to a paucity of specific in-context examples to help educators consider the relevance of the theories to their teaching setting. This scoping review attempts to provide an overview of the use of social theories of learning (SToLs) in HPEPs.

**Method:**

A scoping search strategy was designed to identify the relevant articles using two key concepts: SToLs, and HPEPs. Four databases (PubMed, ERIC, ProQuest, and Cochrane) were searched for primary research studies published in English from 2011 to 2020. No study design restrictions were applied. Data analysis involved a descriptive qualitative and quantitative summary according to the SToL identified, context of use, and included discipline.

**Results:**

Nine studies met the inclusion criteria and were included in the analysis. Only two SToLs were identified in this review: Bandura's social learning theory (*n* = 5) and Lave and Wenger's communities of practice (CoP) theory (*n* = 4). A total of five studies used SToLs in nursing programs, one in medicine, one in pharmacy, and two used SToLs in multi-disciplinary programs. SToLs were predominantly used in teaching and learning (*n* = 7), with the remaining focusing on assessment (*n* = 1) and curriculum design (*n* = 1).

**Conclusions:**

This review illustrated the successful and effective use of SToLs in different HPEPs, which can be used as a guide for educators and researchers on the application of SToLs in other HPEPs. However, the limited number of HPEPs that apply and report the use of SToLs suggests a potential disconnect between SToLs and educational practices. Therefore, this review supports earlier calls for collaborative reform initiatives to enhance the optimal use of SToLs in HPEPs. Future research should focus on the applicability and usefulness of other theories of learning in HPEPs and on measuring implementation outcomes.

**Systematic Review Registration:** https://www.researchregistry.com/browse-the-registry#registryofsystematicreviewsmetaanalyses/registryofsystematicreviewsmeta-analysesdetails/60070249970590001bd06f38/, identifier review registry1069.

## Introduction

Health professions education (HPE) is the field of expertise applied to the education of health care practitioners which caters to the specific requirements of students and is used to develop, implement, and evaluate all aspects of health professions curriculum (1). Acknowledging and understanding the theories behind the learning process is important in optimizing learning environments, enhancing efficiency, and harmonizing the education system (2), since theory and practice are inextricably linked and mutually inform each other (3, 4). Understanding learning theories helps academics and researchers recognize the nature of knowledge acquisition and how to measure learning outcomes. This improved perception will enhance the scholarship of teaching and the understanding of educators within various contexts, namely teaching, curriculum development, mentoring, academic leadership, and learner assessment (5). Furthermore, it will help learners recognize their learning processes and ultimately assist in enhancing their learning outcomes (6). Learning theories can be implemented, based on appropriateness, in learning processes at individual, group or community levels and in various forms of educational activities (7).

In health professional education programs (HPEPs), learning theories are not regularly and consistently implemented, which has resulted in accreditation bodies dictating educational agendas (8), variation in the extent to which learning theories are used in HPEPs, and ultimately a potential disconnect between learning theories, curriculum design, outcome evaluation, and educational practices (9). This is also evidenced by an unfamiliarity among educators inadequately trained to apply theories in a range of contexts with various learner characteristics (5, 6, 10–12). Mukhalalati and Taylor provide an easy-to-use summarized guide of key learning theories used in HPEPs with examples of how they can be applied. The guide aims to assist healthcare professional educators in selecting the most appropriate learning theory to better inform curricula design, teaching strategies, and assessment methods, which in turn reflects on learner experience (13). There is a paucity of literature reviewing the use of learning theories in HPE, the majority of this being generally descriptive, explaining different learning theories and potential HPEP application. For example, little has been reported about the use of learning theories or active learning strategies in e-learning for evidence-based practices (14), or making suggestions for utilizing the conceptual aspects of learning theories in the identification and implementation of effective practices for evaluating teaching practice (15). With a focus on the significance of health professions educators' professional development, using learning theories to enhance teaching skills, particularly in clinical settings (16), the extant literature does not provide clear guidance, for example via the provision of examples of practical application and how these conceptual frameworks might advance the scholarship of teaching and learning in HPEPs. Therefore, health professions education scholars recommend conducting more research into the influence of implementing learning theories on core education components of the HPEPs, namely: curriculum design, content development, teaching, and assessment (13, 17). Such research aims to demonstrate the benefits of implementing learning theories and pedagogies in HPEPs, ultimately reducing the gap between learning theories and educational practices (17).

*Social theories of learning* (SToLs) play an important role in the design and implementation of HPEPs (2, 10, 18). SToLs integrate the concept of behavioral modeling and focus on social interaction, the person, context, community, and the desired behavior as the main facilitators of learning (19). The use of SToLs in HPEPs varies possibly due in part to a lack of awareness of available SToLs and a paucity of specific in-context examples to help educators consider the theories relevant to their teaching situation. SToLs include zone of proximal development, sociocultural theories, Bandura's social learning and social cognitive theories (SLT and SCT), situated cognition, and communities of practice (13, 20–25). Zone of proximal development is defined as “the distance between the actual development level as determined by independent problem solving and the level of potential development as determined through problem solving under adult guidance or in collaboration with a more capable peer” (26). According to sociocultural theories, learning and development are embedded within social events and take place as a learner interacts with other people, things, and events in a collaborative setting (26). Bandura's social learning theories (SLTs), i.e., SLT and SCT, stress the necessity of observing, modeling, and mimicking other people's behaviors, attitudes, and emotional reactions such that environmental and cognitive variables interact to impact human learning and behavior (18, 25). Situated cognition theory asserts that learning occurs when a learner is doing something in both the real and virtual worlds, and hence learning takes place in a situated activity with social, cultural, and physical settings (27). Community of practice “are groups of people who share a concern or a passion for something they do and learn how to do it better as they interact regularly” (28).

To date, no study has examined the application of SToLs in HPEPs and the nature of their use. Consequently, this scoping review aims to examine the application of SToLs in HPEPs. The specific objectives are to (1) identify the SToLs applied to HPEPs, and (2) examine how SToLs are applied to learning and teaching processes in HPEPs.

## Method

### Protocol and registration

This study adopted a scoping review approach involving exploring and documenting the breadth of knowledge and practice in the investigated topic (29). The protocol for this scoping review was registered at RESEARCH REGISTRY [https://www.researchregistry.com/browse-the-registry#registryofsystematicreviewsmeta-analyses/registryofsystematicreviewsmeta-analysesdetails/60070249970590001bd06f38/] with the number [reviewregistry1069]. This scoping review is compliant with the PRISMA statement for scoping reviews (PRISMA-ScR) (30).

### Eligibility criteria

The main focus of this review was to identify articles that describe the applications of SToLs in undergraduate or postgraduate teaching and learning processes. The eligibility criteria included primary research studies that were electronically available in their entirety, published in English during the last 10 years (i.e., 2011–2020), and that reported the use of a SToL, namely: zone of proximal development, sociocultural theories, Bandura's SLTs, situated cognition, and communities of practice.

Primary research articles should report the use of SToLs explicitly and as a central theme, and a description of how SToLs were applied in HPEPs should be mentioned in order to be included in the study. No restrictions were applied to the study design. Primary research studies that used a theory other than the determined ones, mentioned SToLs only in the introduction, or used SToLs for data analysis, and/or as a theoretical framework, rather than as an intervention or an application in HPEP teaching and learning processes, were also excluded. Moreover, articles published more than 10 years ago were not included. Based on the authors experience in this field and on their extensive review of the literature, the scarcity of research that applies SToLs to undergraduate and postgraduate HPE became apparent (8, 9, 31). An initial testing search was conducted with no timeframe boundaries, to refine the search strategy and conduct a comprehensive review. Despite returning a significant number of records, initial screening indicated the irrelevance of the vast majority of studies. Therefore, the authors decided to restrict the timeframe to 10 years to reflect the most recent application of SToLs in HPE and the growth and volume of knowledge related to teaching and learning. Article types other than primary research literature (e.g., reviews, editorials, letters, opinion articles, commentaries, essays, preliminary notes, pre-print/in process, and conference papers) were also excluded from this review because such applications are usually reported in primary research articles. Theses and dissertations were also excluded because they risked being less scientifically rigorous due to a lack of peer-review and being unpublished in commercial journals (32).

### Information sources

The search strategy was developed by a multidisciplinary team. This included academics (BM, FH, ME, and SE) with expertise in pharmacy, healthcare professions education, learning theories, and systematic review studies, and an academic research and instruction librarian (AB) with expertise in health science, education, pharmacy, and medical databases. A search of the electronic literature was performed by AB in December 2020 and January 2021, using PubMed, ERIC, ProQuest, and Cochrane databases. Two key concepts (SToLs, HPEPs) were combined using the Boolean connector (AND). Keywords used in the social learning theories concept search included: “social learning theories,” “social theories of learning,” “social cognitive theories,” “zone of proximal development,” “sociocultural theories,” “situated cognition,” “community/communities of practice.” Keywords for this concept were combined using the Boolean connector (OR). Keywords used to search for the HPEPs concept included “healthcare professional education,” “health care professional education,” “medical program education,” “pharmacy program education,” “health sciences program education,” “nursing program education,” “midwifery program education,” “nutrition program education,” “dietician program education,” “biomedical program education,” “physiotherapy program education,” “physical therapy program education,” “occupational therapy program education,” “radiation therapy program education,” “public health program education,” and “dental program education.” Keywords for this concept were combined using the Boolean connector (OR). Keywords were matched to database-specific indexing terms and applied based on each database as appropriate.

### Search

The PubMed database was searched on December 22, 2020, implementing date (i.e., 2011–2020) and language (i.e., English only) filters, resulting in 689 articles. The following search strategy was used: “social learning theor^*^”[Title/Abstract] OR “social theor^*^ of learning”[Title/Abstract] OR “social cognitive theor^*^”[Title/Abstract] OR “zone of proximal development”[Title/Abstract] OR “situated cognition”[Title/Abstract] OR “sociocultural theor^*^”[Title/Abstract] OR communit^*^ of practice[Title/Abstract] AND Education[MeSH Terms] OR healthcare professional education[Title/Abstract] OR health care professional education[Title/Abstract] OR health sciences program education[Title/Abstract] OR nutrition program education[Title/Abstract] OR diet^*^ program education[Title/Abstract] OR biomedical program education[Title/Abstract] OR physiotherapy program education[Title/Abstract] OR physical therapy program education[Title/Abstract] OR occupational therapy program education[Title/Abstract] OR radiation therapy program education[Title/Abstract]. Completed search strategies for other databases are presented in Supplementary material 1.

### Selection of sources of evidence

Two investigators (BM and FH) conducted the title and abstract screening for the identified articles from the search strategy outlined above after duplicates and any clearly irrelevant articles had been removed. Full-text screening was conducted initially by two investigators (BM and MJ) who assessed the eligibility of the studies independently. Further multiple rounds of full-text reviews were performed by four investigators (BM, SE, MJ, and FH) to ensure that studies directly relevant to the objectives were included in this review. Disagreements were resolved by consensus via meetings and discussions.

### Data charting process and data items

An extraction sheet was designed to tabulate data from the included articles using a Microsoft Excel^®^ spreadsheet. The extracted data included: (1) article information (title, author(s), year of publication, and journal name), (2) setting information (setting, organization name, whether the organization was public or private, and country), (3) research information (objective, design, HPEP), (4) theory information (name of the theory, context of application, description of how the theory was applied, the outcomes assessed, and methods of analysis), (5) outcome information (number of participants involved, intervention provided, duration of intervention, overall outcome and recommendations, and reported limitations related to the theory), and (6) the applicability to other disciplines. The context of the SToLs application includes teaching and learning (strategies used to deliver and receive educational content in clinical or non-clinical settings, etc.), curriculum development (learning objectives, planning of teaching strategies, program evaluation, etc.), or assessment (development, validation, and administration of assessment activities, etc.). The data extraction sheet was piloted by two investigators (BM and MJ) using four sample articles included in this review. Based on successful piloting, complete data extraction was done by four investigators (BM, FH, ME, and SE).

### Critical appraisal of individual sources of evidence

The included studies were not evaluated for quality or critically appraised because of methodological heterogeneity among studies. However, this lack of quality evaluation and critical appraisal aligns with the general standards of scoping reviews (33).

### Synthesis of results

Descriptive numeric analysis was used to summarize data retrieved from the included articles according to the proportion of (1) articles per discipline, (2) SToLs applied, and (3) contexts in which SToLs were used. Moreover, the analysis of the data involved conducting a narrative description of the included articles by two independent investigators (MJ and FH). Consensus was reached on the basis of the analyzed data.

## Results

**Figure 1:**
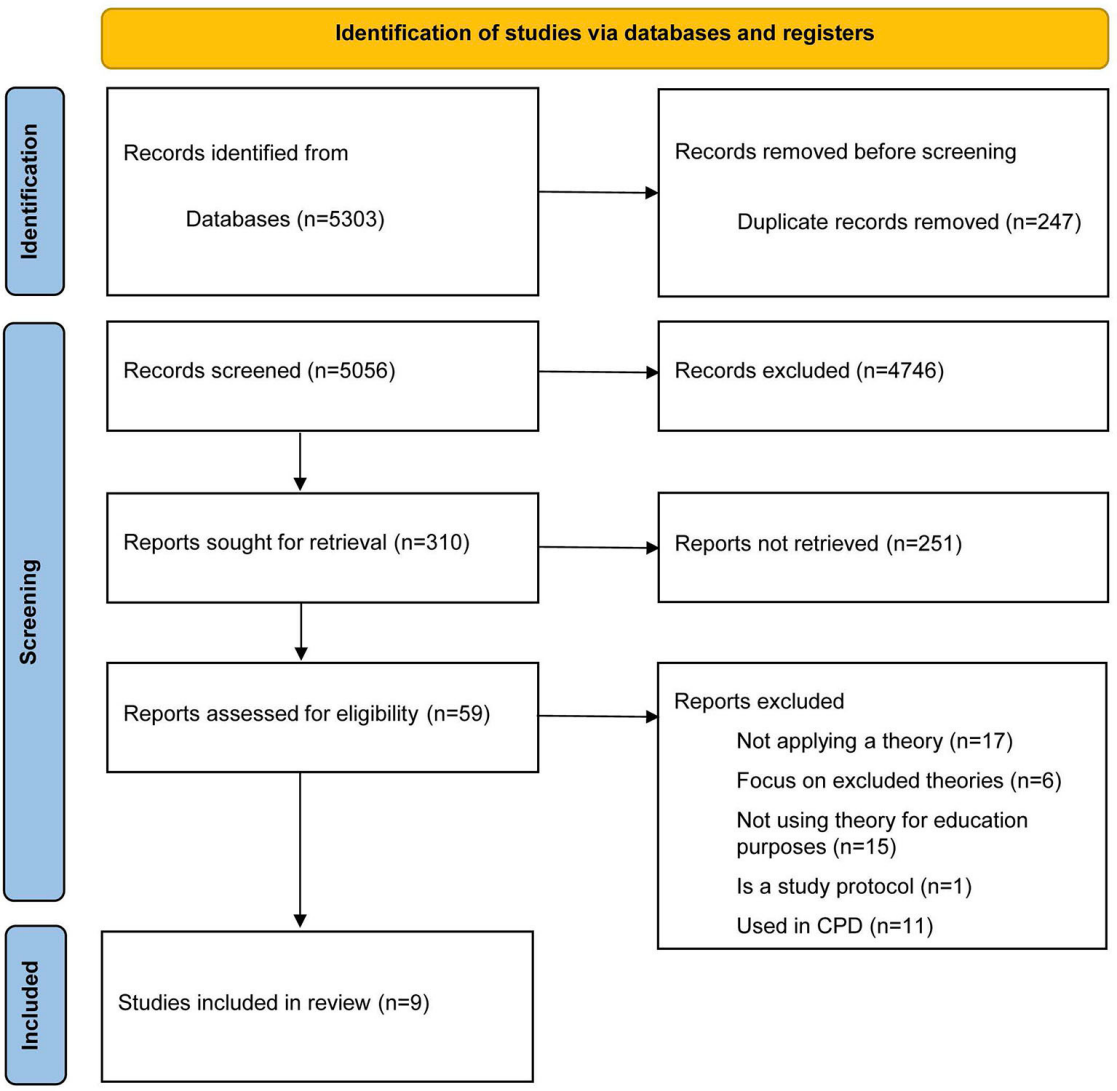
Prisma flow diagram.

Out of 5,303 articles retrieved from databases, 247 were duplicates and hence removed (Figure 1). Following the title and abstract screening of 5,056 articles, 310 articles were eligible for full-text screening. Primary reasons for exclusion include: article types other than primary research literature (e.g., review articles, description of a theory, editorial letters, commentaries, protocols), thesis or dissertations, articles that described the use of theories other than SToLs, articles that did not implement SToLs or did not implement them in undergraduate or postgraduate education (e.g., implemented them for faculty development), articles that focused on other professions and not on health professions, and articles that used SToLs for data analysis purposes. Other reasons for exclusion included manually detected duplicates. A total of nine articles were qualified for inclusion and were used to inform this scoping review.

### Characteristics of included studies

Of studies published between 2013 and 2019, two studies were conducted in the USA (34, 35), three in Australia (36–38), and one study in each of these countries: Sweden (39), Canada (40), Scotland (41), and Italy (42). A total of five studies used Bandura's SLTs (34, 36–38, 40), while four used Lave and Wenger's CoP theory (35, 39, 41, 42). Three studies used a qualitative research methodology (39–41), two studies used quantitative research methodology (37, 42), and three studies used a mixed-method design (35, 36, 38). The remaining study, educational innovation, focused on describing the implementation of a teaching strategy (34). A total of five studies used SToLs in nursing programs (34, 38, 40–42), one in medicine (39), one in pharmacy (37), and two were multi-disciplinary, including: paramedicine, psychology, nutrition and dietetics, nursing, public health, medicine, and other HPEPs (35, 36). Seven studies used *SToLs in teaching and learning* (34, 36–39, 41, 42)*, one in assessment* (40), *and one in curriculum design* (35). The included studies covered a total of 1,780 participants (i.e., undergraduate students, residents, clinical teachers, and healthcare professionals) (Table 1).

**Table 1:** Characteristics of the included studies.

**Author year country setting**	**Objective**	**Design**	**Level professions (*n*)**	**Theory used application and intervention duration**	**Overall study outcomes/effectiveness of the intervention**	**Limitations strengths recommendations**
Carroll et al. (36) Australia University	To explore engagement of students to response systems such as GoSoapBox and explain its contribution to the learning process	Explanatory Sequential mixed method	Undergraduate Paramedic, psychology, nutrition, dietetics, nursing, and public health (*n* = 350)	SLT in teaching Implemented the four stages of observational learning of Bandura's SLT • Attention: students initially watched and assessed the interactions and contributions of the cohort leaders • Retention: more critical evaluations were made about what was working and what was not • Reproduction: participation in the discussion was encouraged • Motivation: expose to a variety of models which are not available in traditional classroom format. Duration: one semester	The use of SLT in investigating the effectiveness of GoSoapBox proved that it is a valuable tool for stimulating conversations and debates on controversial topics, such as gender, religion, and politics The SLT framework found that students gained the ability to participate in discussions which may lead to sustained learning and improved critical thinking	• Although anonymity encouraged discussion, but also created unsafe learning environment for marginalized students • Discussion component of GoSoapBox was the best component to students' learning, followed by Polls and social questions and answers. • GoSoapBox use requires a code of conduct outlining appropriate behavior to ensure safe spaces, minimize distraction, and increase learning
Carter et al. (37) Australia University	To develop and test a model, based on SCT, of final-year students' intending to undertake a higher degree in PPR after graduation	Quantitative questionnaire	Undergraduate Pharmacy (*n* = 386)	SCT in teaching and developing a model A hypothesis was generated from SCT which suggested that a person's motivation to undertake a particular activity may be influenced by their self-efficacy and outcome expectancy Final year students in the final week of the semester undertook a survey to investigate interest in Pharmacy practice research after graduation Duration: 1 week	Pharmacy practice educators have role in influencing students' undertaking PPR as a career Exposure to PPR appears to have little influence on students' perceptions of PPR as a career To increase pharmacy students' selection of PPR as a career path, pharmacy practice educators need to provide links between research and practice	• Used a structural efficacy model to test the hypothesis in a cross-sectional study Generalizability of findings is limited The direction of influence between self-efficacy and outcome expectancy requires some consideration Convergent validity is absent • Mentoring programs in PPR are recommended Longitudinal study is warranted
Irvine et al. (38) Australia University	To determine SRL strategies used by final year students.	Concurrent mixed methods study (questionnaire and interviews)	Undergraduate Nursing students (*n* = 319)	SCT and self-regulated learning in teaching SRL is a learning model situated in SCT and considers learners as active participants in their learning process with the ability to monitor, manage, and regulate specific parts of their cognition, motivational behaviors, and surroundings • Used a questionnaire that reliably measures the 15 scales of Pintrich's social cognitive model of SRL In the qualitative part, an analysis protocol was used, a theory guided approach, using question prompts linked to theoretical categories of SRL Duration: one semester	High levels of motivational and learning strategies were used by students in their approach to learning, and in their roles as near-peer teachers Learning strategies were associated with higher- order learning A dyadic approach in peer teaching can support metacognitive-shared regulation and identify how self-doubt may affect NPTs' performance	• Limited generalizability and data integration • Significance of incorporating SRL in the undergraduate nurse curriculum to enhance students' performance and promote confidence in their future teaching opportunities in clinical settings
Kennedy et al. (40) Canada University	To develop and psychometrically assess the Nursing Competence Self-Efficacy Scale (NCSES)	Quantitative questionnaire	Undergraduate Nursing students (*n* = 252)	Self-efficacy and SCT in assessment A 22 item NCSES was developed to measure nursing students' self-efficacy for practice competence based on Bandura's SCT theory: • The wording in the stem of each item used phrases that are concerned with the perceived capabilities and not with the intention • Used a 9-point response format to increase discrimination, Duration: 6 weeks	A scale with evident construct validity, internal consistency reliability, and test–retest stability reliability Can be used to examine undergraduate nursing students' self-efficacy practice competence, assist educators in determining the level of education that students receive, as well as assess novel curriculum interventions targeted at improving students' self-efficacy	• Relevance of the NCSES in other countries is not yet determined • Valid and reliable scale • Further psychometric assessment of the scale is warranted Qualitative studies in relation to curriculum initiatives or adaptations based on SCT, will increase the current understanding of the construct of interventions targeted at improving students' self-efficacy
Koo et al. (34) USA University	To develop a formative standardized patient experience.	Descriptive study	Undergraduate Nursing students (*n* = 30)	Self-efficacy and SCT in teaching Used to guide the development of simulated clinical experiences to allow learners to develop collaborative self-efficacy by sequentially participating in two simulated clinical scenarios. Students participated in observational learning by seeing their classmates participate in these two scenarios as inter-professional teams Duration: not mentioned, but was completed in the final semester	Students' self-efficacy was developed through incremental mastery experiences by repeating the clinical scenarios on more than one occasion Problem-solving and communication skills, and clinical competency were improved Interdisciplinary collaboration and IPE were promoted	• No objective assessment was conducted • This intervention can be utilized as a teaching tool to develop IPE that can be replicated in a simulated clinical setting and facilitate collaborative practices among health professional students and faulty • learning objectives and simulation scenarios needed to be revised • Facilitating faculty, standardized patients, and collaborating professionals should have adequate training for the scenarios and provide constructive feedback to students
Alsiö et al. (39) Sweden Hospital and academia	To explore HCP experiences of implementing clinical education of medical students in CoP	Qualitative research (Focus groups)	Practice Assistant nurses, nurses and physicians (*n* = 35)	CoP in teaching Creating teams to enhance student engagement, participation in practice education and to develop learning activities for the students informed by set learning objectives to reach a common goal Duration: not mentioned	CoP stimulate individual learning, and enhance clinical work Implementing student education at a hospital stimulate learning among staff and was effective for structural development in CoP Opportunities for inter-professional interaction and reflection are vital to successfully implement a new student in CoP	• Study conducted in one healthcare context • Rich and trustworthy data generated from the focus groups • The need for clinical education opportunities in many countries is increasing. Therefore, the support for staff engagement when implementing education of medical students in CoP needs to be explored
Molesworth et al. (41) Scotland University	To explore how students perceive biosciences in the curriculum.	Qualitative focus groups and interviews	Undergraduate Nursing students (*n* = 7)	CoP in teaching Understanding students' perceptions of how bioscience education is used in practice during their clinical placements which represents CoP. Students were interviewed after the first and second year of being involved in CoP Duration: 2 years	Three themes emerged: Bioscience learning within practice, incorporating bioscience knowledge into practice and bioscience knowledge and perceived competence Authors recommend using CoP (practice setting) to reinforce and teach students the biosciences (theory)	• A study that is limited in scale • Shine a light on students' perspectives of bioscience in practice • Research is required into the role and effectiveness of bioscience-related learning within practice settings
Portoghese et al. (42) Italy University	To expand the knowledge of the CoP in the healthcare setting by analyzing students' perception of respect they were shown during their clinical placements	Quantitative questionnaire	Undergraduate Nursing students (*n* = 188)	CoP in teaching The clinical practice component of nurse education programs represents an example of a CoP setting where nursing students acquire and advance in the knowledge and skills of nursing CoP was used to describe the practice setting and understand the student experiences Duration: not mentioned	Feedback and support received from members CoP, and quality of student-tutor relationship showed significant effects on students' perceived respect Social situation might influence students' perceptions of respect while examining nursing students in a CoP	• Lack of questionnaire validation Limited generalizability Cross-sectional study • Use of quantitative data in CoP research • Longitudinal-type investigation is needed to observe changes of the students' perceptions relating to the role of CoP as working and learning context for clinical practice
Chen et al. (35) USA University	To describe the HPE Pathway program development, curriculum, and initial program outcomes by focusing on the pathway's CoP approach to supporting career development of students as future educators	Mixed method (quantitative program evaluation and qualitative email survey)	Undergraduate and Practice medical students, residents, and fellows, learners from other HPE schools. (*n* = 71)	CoP in teaching through course requirements, learners engage and work with members of the educator CoP. Pathway instructors (health professions educators) are faculty members who model a breadth of educator careers to help learners imagine personal trajectories. Then learners completed mentored educational projects Duration: 5 years	Learners gained knowledge and skills for continued engagement with CoP educators, confirmed their career aspirations, joined an educator-in-training community (engagement/imagination), and disseminated via scholarly meetings and peer-reviewed publications (alignment) Learners identified engagement with the learner community as the most powerful aspect of the pathway.	• HPE Pathway provides a robust example of employing a CoP framework to developing health professions educators

*CoP, Community of Practice; HCP, Healthcare professionals; HPE, Health professions education; SCT, Social cognitive theory; SLT, Social learning theory; SRL, Self-regulated learning; NCSES, Nursing Competence Self-Efficacy Scale; NE, Nutrition Educator; NPT, Near peer teaching; PPR, Pharmacy practice research; RN, Registered nurses*.

### Bandura's SLTs

Five studies in this scoping review focused on utilizing Bandura's SLTs in the teaching, learning, and assessment of health professions students (34, 36–38, 40). The use of Bandura's SLTs in the included studies suggested its advantages in improving students' self-efficacy and confidence, collaborative learning, learning experiences and future teaching experience and career research intentions.

In 1977, Bandura proposed an SLT based on a series of human behavioral studies (24). According to Bandura, learning takes place in social settings and occurs not only through an individual's own experiences, but by observing the actions of others and their consequences (24, 43). Social learning is also referred to as observational learning because learning takes place as a result of observing others (i.e., models), which Bandura's previous studies demonstrated as a valuable strategy for acquiring new behaviors (44). Bandura and his colleagues continued to demonstrate modeling/observational learning as a very efficient method of learning (44). Bandura's theorizing of the social development process later incorporated motivational and cognitive processes into SLT (44). In 1986, Bandura renamed his original SLT to SCT to emphasize the critical role that cognition plays in encoding and the performance of activities (44, 45). SCT suggests that learning occurs in a social context with a dynamic and reciprocal interaction of the person, environment, and behavior (25). The core constructs of SCT include modeling/observational learning, outcome expectancies, self-efficacy and self-regulation (25, 44). Bandura's observational learning consists of four stages: (1) attention: learners see the behavior they want to reproduce, (2) retention: learners retain the behavior they have seen entailing a cognitive process in which learners mentally rehearse the behavior they wish to replicate, (3) reproduction: learners put the processes obtained in attention and retention into action, and (4) motivation: learners imitate the observed behavior through reinforcement (direct, vicarious or self-reinforcement).

Based on Bandura's argument that human behavior is learnt via interactions with, and modeling of others in social contexts, Carroll et al. (36) applied the four stages of observational learning to investigate the effectiveness of GoSoapBox, a student response system (SRS). The study proved the effectiveness of this online tool in stimulating discussions on controversial topics, improving learning experiences and in-class engagement among paramedic, psychology, nutrition and dietetics, nursing, and public health students.

Carter et al. (37) focused on the self-efficacy, outcome expectancy and social influence components of SCT to develop and test a model that evaluates undergraduate pharmacy students' intentions to pursue a higher pharmacy practice research (PPR) degree. The authors suggest that educators must provide links between practice and research and increase student self-confidence to undertake PPR, thereby increasing interest in this as a future career path. This is because exposure alone has minimal influence on a student's interest in PPR as a career.

Irvine et al. (38) explored self-regulated learning (SRL), a learning model situated in SCT, strategies utilized by final year nursing students in both their approaches to learning and practical teaching sessions (peer-teaching). The study findings support the use of SRL in nursing education, as highlighted by the high level of motivational behaviors and learning strategies reported among undergraduate nursing students in their approach to learning and their roles as peer-teachers.

Kennedy et al. (40) used the construct of self-efficacy to develop and psychometrically assess a scale that examines undergraduate nursing students' self-efficacy practice competence, assist educators in determining the level of education that students receive, as well as their level of confidence and advocacy for positive changes.

Furthermore, Koo et al. (34) indicated that implementing a self-efficacy construct to develop a formative standardized patient experience allowed nursing students to develop the concepts of inter-professional collaborative communication, and enhanced their problem-solving and communication skills, as well as their clinical competency.

### Lave and Wenger's theory: Communities of practice (CoP)

The CoP theory consists of three key components: the domain (the common interest among all members), the practice (the implicit and explicit knowledge shared), and the community (made up of mutually beneficial interactions between experts and learners leading to learning, engagement, and identity development) (10, 46–48). All the articles retrieved in this review described a CoP as a group of people who share similar characteristics and collaborate toward a common goal, therefore enhancing mutual learning through sharing relevant knowledge and fostering the development of a shared identity. Three of the studies implemented CoP theory with a focus on teaching and learning among health professions students, and one with a focus on HPEP curricula design. All studies indicated that implementing the CoP learning theory enhanced student learning, collaboration, and identity.

Alsio et al. (39) found that when CoP theory was used to create teams of practicing nurses, physicians, and undergraduate medical students with the mandate of developing learning activities during their clinical placements, learning was stimulated through self-reflection and consideration of their perspectives during patient interactions. Further, inter-professional reflection was vital for successful introduction of new students into a CoP and was effective for structural and cultural development. Moreover, staff and students' awareness of their roles and responsibilities facilitated their motivation to participate in the CoPs implementation.

Similarly, Molesworth et al. (41) and Protoghese et al. (42) explored the experiences of undergraduate nursing students regarding their application of the CoP theory during clinical placements. Both studies argued that CoP helped students to integrate their theoretical learning of bioscience into practice (41), and to advance their existing clinical knowledge (42). Moreover, application of bioscience knowledge within a CoP facilitated effective inter-professional relationships (41). Additionally, students perceived that they received more respect, support, and feedback while learning within a CoP (42). This further emphasizes the significance of mutual engagement and the collaborative relationship component of the CoP theory in enhancing student learning (42).

Furthermore, Chen et al. (35) used CoP theory in a curricular design for the HPEP aimed at helping undergraduate medical students, residents, fellows, and learners from other HPE schools to develop their identities as future health professions educators. The program has demonstrated its effectiveness in providing learners with the knowledge and skills to realize their career aspirations. It also enhanced learners' enthusiasm for teaching and increased their interest in educational leadership, innovation, and research.

## Discussion

This scoping review attempted to provide an overview of how SToLs have been used in the teaching and learning of HPEPs over the last decade. This review highlighted some interesting findings that, collectively, may provide insights into how educational practices in HPEPs are shaped and influenced by learning theories.

### Bandura's SLTs

Bandura's SLTs were applied predominantly in teaching and instruction strategies within the HPEPs. This review demonstrated the application of Bandura's observational learning model in the form of in-class integrated collaborative learning activities through an online tool for improving learning experiences and engagement (36). It is argued that observational learning provides a faster and safer approach to learning complicated patterns of behavior than trial and error, making it consistent with and suitable for HPE (7, 49). Self-efficacy, defined by an individuals' assessment of their capacity to perform given tasks or activities and achieve specified goals (50), was the most highlighted construct in the included articles. This can be explained by Bandura's argument that self-efficacy is central to social learning because it significantly impacts a wide range of human endeavors, including developmental and health psychology, education, and in the workplace (19). The findings suggest that the self-efficacy construct is beneficial to the learning outcome, particularly in simulation contexts, as demonstrated in the review conducted by Lavoie et al. (51). This aligns with previous literature about the self-efficacy construct indicating that individuals with stronger self-efficacy for certain tasks are more motivated to execute them (50, 52). Furthermore, the self-efficacy construct was used to develop an assessment tool that evaluates students competence and confidence level and advocacy for positive changes as they become professional nursing practitioners (40). In this context, it is worth mentioning that assessment tools based on self-efficacy found in previous health-related literature are task-specific (53, 54). Previous literature has also argued that feelings of confidence among medical students are associated with competence and proficiency (55, 56), and lack of confidence leads to nurses leaving the profession (57). Moreover, clinical educators' self-efficacy and confidence are critical to their ability to carry out their teaching and training responsibilities as they affect student achievement and patient outcomes (58).

### Lave and Wenger's CoP theory

In this review, CoP theory was mainly employed in the teaching and learning of health professions students, educators, and providers to improve learning, collaboration, and identity. However, as highlighted by Hörberg et al. (59), it would be better used to identify team challenges and provide more meaningful interventions. It is noteworthy that none of the included studies highlighted any long-term benefits of CoP, aligning with Allen et al.'s (60) argument that there is a paucity of health professions studies exploring the long-term effect of CoP on individuals and the relevance to educational outcomes. Additionally, several studies in healthcare education and practice indicated the scarcity of studies that focus on the development and assessment of CoPs (10, 61, 62).

This review highlights a scarcity of research focusing on the application of SToLs in the development, validation, and conduction of assessment activities within HPEPs. Only one study used the self-efficacy construct to develop a tool for assessing student competence (40). This is consistent with a recent literature review suggesting that SToLs are not applied in performing assessment activities compared to other learning theories, such as humanistic theories or motivational models (13). This is despite evidence of the utility of CoP learning theory in planning and implementing effective assessment measures in the PharmD program (20).

The current review suggests that the application of SToLs in designing HPEPs' curricular content, learning objectives, syllabus or influencing educational competencies is also not common. In this regard, Mukhalalati and Taylor proposed a novel CoP theory-informed framework that can be used in designing a new HPEP to reduce the disconnect between the educational practice and learning theories (10). The authors suggest key components to consider when developing a CoP-based curriculum, including but not limited to, complementing formal with informal learning, transferring tacit knowledge to explicit knowledge through socialization and externalization, re-contextualizing knowledge, and aligning students' learning needs to learning activities (10). These components are compatible with several SToLs and claimed to be applicable in various HPEPs (10).

An important observation in this review was the exclusion of a large number of retrieved articles because they failed to inform how SToLs are implemented in the educational practices and in delivering educational goals (63), or because they aimed to use SToLs as a lens to explore HPEPs teaching and learning practices, or as a theoretical framework to conceptualize or analyze HPE research data (64–66). This aligns with previous research that highlighted the significance of using theories to enhance research rigor and its relevant outcomes (67). However, it is suggested to use learning theories to critique HPE and guide its advancement initiatives (68, 69). Furthermore, several excluded studies utilized SToLs for healthcare professionals continuing professional development (70–75), which seems to be a common application of SToLs. Although examining SToLs utilization in continuing professional development activities was not the aim of conducting this review, this aspect is extremely important as it indirectly influences students who will ultimately become health care professionals. Collectively, the small number of included eligible studies in this review that applied SToLs in HPEPs suggests disconnect between SToLs and HPEPs educational practices. It is argued that it is challenging for HPEPs educators to apply the educational theories because they received minimal or no educational training about their significance and implementation (5). Therefore, as recommended by previous research, a collaborative reform initiative should be enacted to enhance the optimal use of SToLs in educational practice and examine the applicability and usefulness of other theories of learning in HPEP (20). Moreover, this review did not include studies from Africa, Eastern Mediterranean, and South-East Asia, suggesting that exploratory and experimental educational research utilizing various learning theories are highly warranted in these regions.

### Strengths and limitations

This review explored SToLs use in HPEPs and provided a valuable overview for educators in a broad range of health education fields. Studies included were conducted in various countries which further enhanced the results' applicability to other contexts. However, a number of limitations should be acknowledged when interpreting the findings of this review. For example, this review was limited to only four databases and to the last decade, potentially missing relevant articles in other major databases such as Scopus and Web of Science and those published before 2011. Moreover, as is inherent to scoping reviews, a quality assessment for the included articles was not conducted necessitating caution in interpreting conclusions. Additionally, since SToLs can be categorized and named differently, this might inadvertently result in the omission of relevant articles.

## Conclusions

This review provides an overview of the application of SToLs in HPEPs from 2011 to 2020. Only two SToLs were identified in this review: Bandura's SLT and SCT; and Lave and Wenger's CoP theory. Bandura's four-stage model of observational learning, as well as self-efficacy construct, were applied in the included studies. CoP theory was mainly employed to improve learning, collaboration, and identity, whilst SToLs use was predominantly focused on teaching and learning with less focus on assessment and curriculum design. This review demonstrated a limited number of HPEPs applying and reporting an application of SToLs despite the significance of the social aspect of learning concepts in those theories and within HPEP. This suggests a potential disconnect between SToLs and HPEP educational practices. Nonetheless, this review illustrated the successful and effective implementation of StoLs in various HPEPs, which is applicable to other HPEPs. Finally, this review supports the call for collaborative reform initiatives to optimize the use of StoLs in HPEPs educational practices. Future research should focus on the applicability and usefulness of other theories of learning in HPEP and investigate the long-term outcomes of theory implementation.

## Data availability statement

The original contributions presented in the study are included in the article/Supplementary material, further inquiries can be directed to the corresponding author/s.

## Author contributions

All authors listed have made a substantial, direct, and intellectual contribution to the work and approved it for publication.

## Funding

Open Access funding is provided by the Qatar National Library.

## Conflict of interest

The authors declare that the research was conducted in the absence of any commercial or financial relationships that could be construed as a potential conflict of interest.

## Publisher's note

All claims expressed in this article are solely those of the authors and do not necessarily represent those of their affiliated organizations, or those of the publisher, the editors and the reviewers. Any product that may be evaluated in this article, or claim that may be made by its manufacturer, is not guaranteed or endorsed by the publisher.
