# Supplementary material for: Applications of social theories of learning in health professions education programs: A scoping review
Source: Front Med (Lausanne). 2022 Jul 28;9:912751. doi: 10.3389/fmed.2022.912751 (PMC9367215; doi:10.3389/fmed.2022.912751)
Supplement: Supplementary file 1 [file Table_1.DOCX]

## Supplementary Materials

**Supplementary Material 1. Search Strategies for ProQuest, Eric and Cochrane databases**

| **Database** | **Search Strategy** | **Number of Results** | **Filers** | **Date** |
| --- | --- | --- | --- | --- |
| ProQuest | TIAB("social learning theories") OR TIAB("social theories of learning") OR TIAB("social cognitive theories") OR TIAB("zone of proximal development") OR TIAB("sociocultural theories") OR TIAB("situated cognition") OR TIAB("community/communities of practice") OR TIAB(constructivism) OR TIAB("experiential learning theories")  AND  TIAB(education) OR TIAB(healthcare professional education) OR TIAB(health care professional education) OR TIAB(medical program education) OR TIAB(pharmacy program education) OR TIAB(health sciences program education) OR TIAB(nursing program education) OR TIAB(midwifery program education) OR TIAB(nutrition program education) OR TIAB(diet* program education) OR TIAB(biomedical program education) OR TIAB(physiotherapy program education) OR TIAB(physical therapy program education) OR TIAB(occupational therapy program education) OR TIAB(radiation therapy program education) OR TIAB(public health program education) OR TIAB(dental program education) | 240 | English  Last 10 years | January 6, 2021 |
| ERIC | TI social learning theor* OR AB social learning theor* OR TI social theor* of learning OR AB social theor* of learning OR TI social cognitive theor* OR AB social cognitive theor* OR TI zone of proximal development OR AB zone of proximal development OR TI sociocultural theor* OR AB sociocultural theor* OR TI situated cognition OR AB situated cognition OR TI communit* of practice OR AB communit* of practice OR TI constructivism OR AB constructivism OR TI experiential learning theor* OR AB experiential learning theor*  AND  TI education OR AB education OR TI healthcare professional education OR AB healthcare professional education OR TI health care professional education OR AB health care professional education OR TI medical program education OR AB medical program education OR TI pharmacy program education OR AB pharmacy program education OR TI health sciences program education OR AB health sciences program education OR TI nursing program education OR AB nursing program education OR TI midwifery program education OR AB midwifery program education OR TI nutrition program education OR AB nutrition program education OR TI diet* program education OR AB diet* program education OR TI biomedical program education OR AB biomedical program education OR TI physiotherapy program education OR AB physiotherapy program education OR TI physical therapy program education OR AB physical therapy program education OR TI occupational therapy program education OR AB occupational therapy program education OR radiation therapy program education OR AB radiation therapy program education OR TI public health program education OR AB public health program education OR TI dental program education OR AB dental program education | 3616 | English  Last 10 years | January 7, 2021 |
| Cochrane | (social learning theories):*ti,ab* OR (social theories of learning)*:ti,ab* OR (social cognitive theories) *:ti,ab* OR (zone of proximal development)*:ti,ab* OR (sociocultural theories)*:ti,ab* OR (situated cognition)*:ti,ab* OR (community of practice)*:ti,ab* OR (constructivism)*:ti,ab* OR (experiential learning theories)*:ti,ab*  AND  (education)*:ti,ab* OR (healthcare professional education)*:ti,ab* OR (health care professional education)*:ti,ab* OR (medical program education)*:ti,ab* OR (pharmacy program education)*:ti,ab* OR (health sciences program education)*:ti,ab* OR (nursing program education)*:ti,ab* OR (midwifery program education)*:ti,ab* OR (nutrition program education)*:ti,ab* OR (diet program education)*:ti,ab* OR (biomedical program education)*:ti,ab* OR (physiotherapy program education)*:ti,ab* OR (physical therapy program education)*:ti,ab* OR (occupational therapy program education)*:ti,ab* OR (radiation therapy program education)*:ti,ab* OR (public health program education)*:ti,ab* OR (dental program education)*:ti,ab* | 724 | English  Last 10 years | January 17, 2021 |
